# Supplementary figures and images for: Combining genomics and epidemiology to track mumps virus transmission in the United States
Source: PLoS Biol. 2020 Feb 11;18(2):e3000611. doi: 10.1371/journal.pbio.3000611 (PMC7012397; doi:10.1371/journal.pbio.3000611)

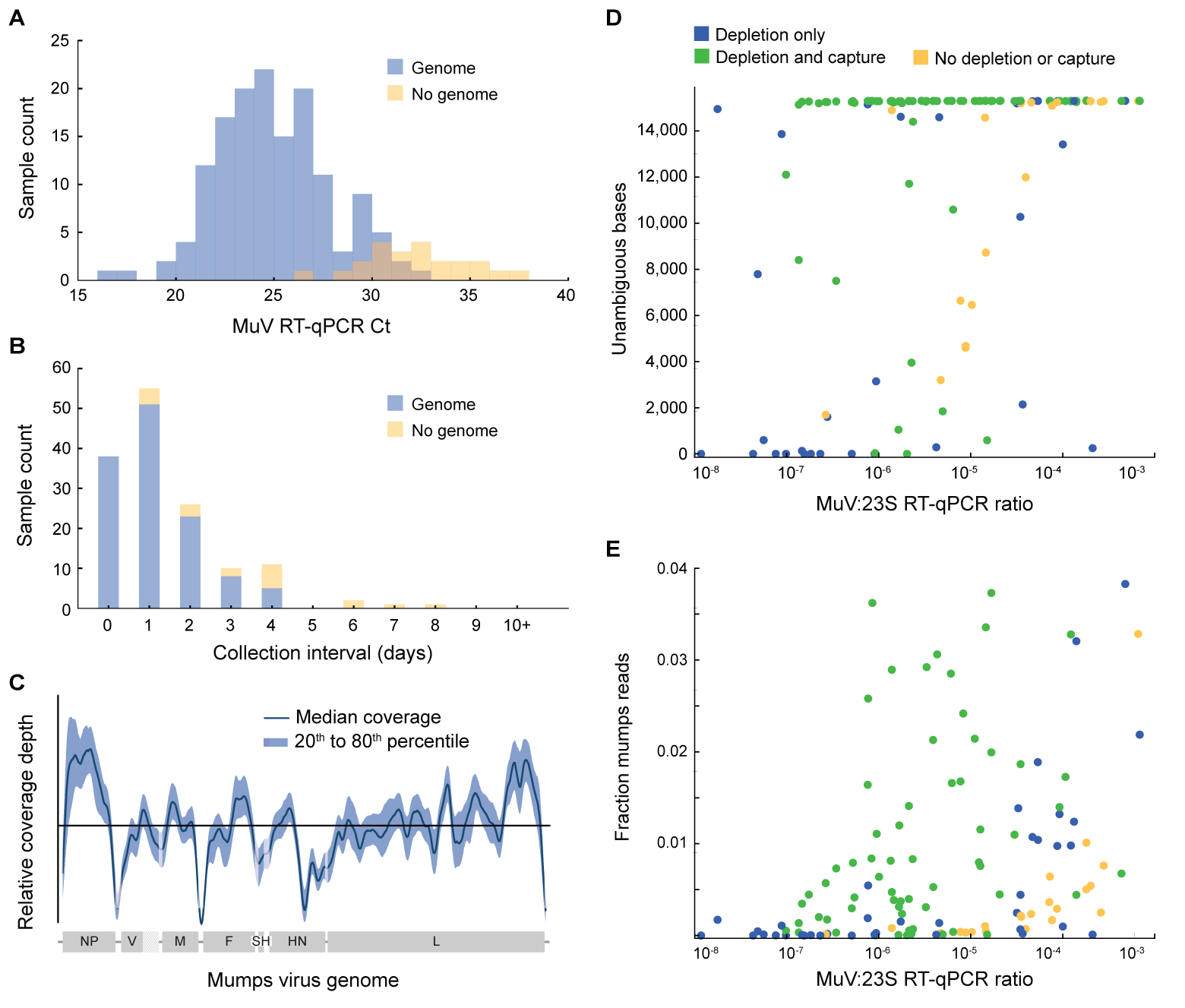

Supplement: S1 Fig — (A) Distribution of mumps virus (MuV) RT-qPCR Ct value, taken at sample source, for all sequencing replicates prepared with both depletion and capture (see Materials and methods). Genome (blue): a replicate produces a genome passing the thresholds described in Materials and methods. MuV RT-qPCR serves as a predictor of sequencing outcome. (B) Distribution of collection interval (days between symptom onset and sample collection) for all samples prepared with both depletion and capture. Genome (blue) is defined as in panel A. Samples taken more than 4 days after symptom onset did not produce genomes in this study [88]. (C) Relative sequencing depth of coverage aggregated across 203 mumps genomes. (D) Number of unambiguous bases in the genome assembly of each sample by MuV:23S ratio (MuV copies by MuV RT-qPCR divided by 23S copies by 23S RT-qPCR; see Materials and methods). Each point is a replicate, colored by sequencing preparation method. (E) Normalized MuV reads (unique MuV reads divided by raw sequencing depth) in each sample by MuV:23S ratio. Points are as in panel D. Nine points with fraction mumps reads >0.04 are beyond the y-axis limits. In panels A, B, D, and E, reads from each replicate were downsampled to 1 million prior to assembly (see Materials and methods). In panels D and E, 1 point with a MuV:23S ratio <10−8 and 3 points with a MuV:23S ratio >10−3 are beyond the x-axis limits. Ct, cycle threshold; MuV, mumps virus; RT-qPCR, real-time quantitative polymerase chain reaction. (TIF) [file pbio.3000611.s001.tif]

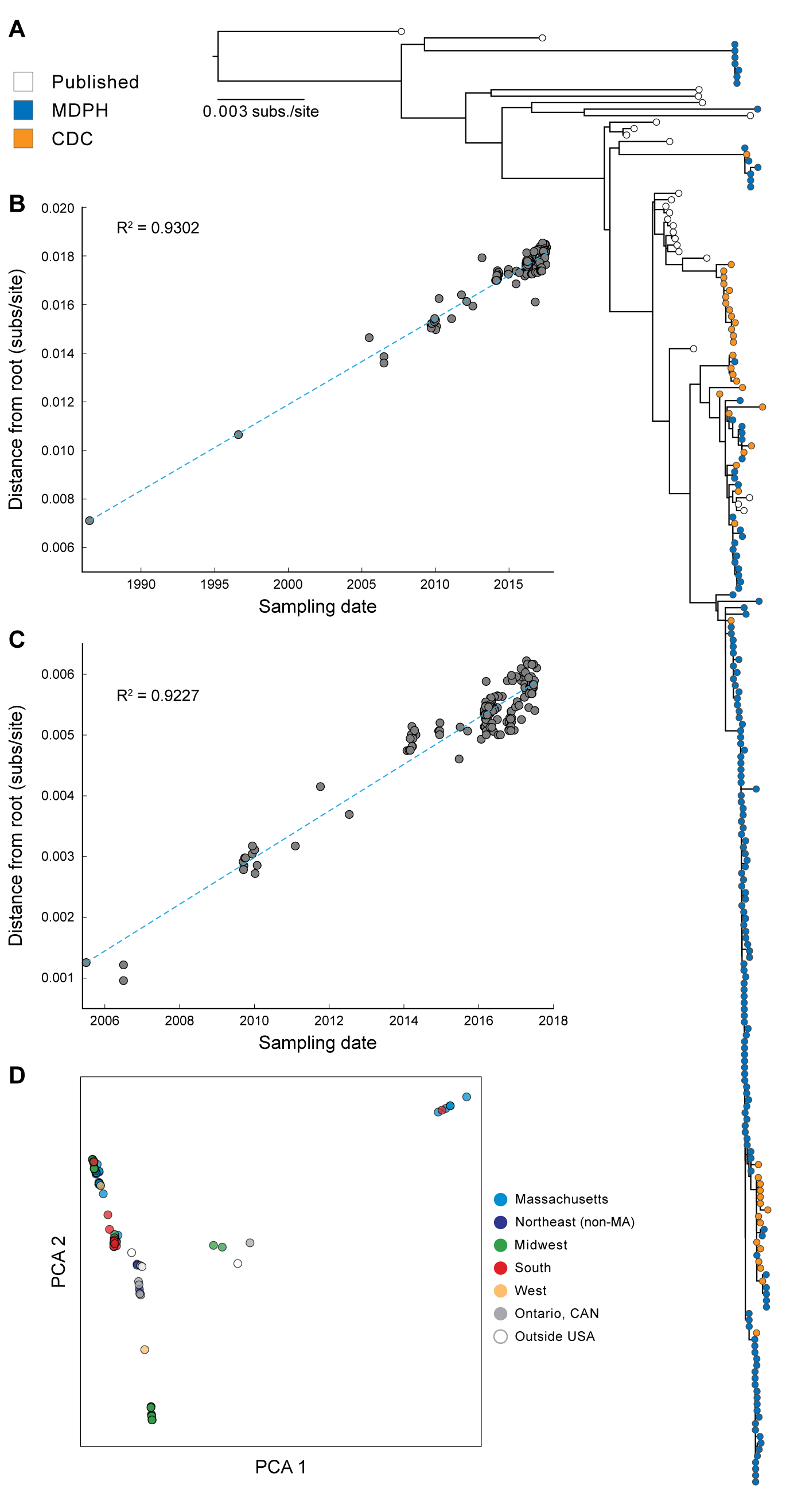

Supplement: S2 Fig — (A) Maximum likelihood tree of the 225 mumps virus genotype G genomes used in this study. Tips are colored by sample source (MDPH or CDC); previously published genomes are indicated by unfilled circles. (B) Root-to-tip regression of genomes shown in panel A, rooted on GenBank accession KF738113 (Pune.IND, 1986). (C) Root-to-tip regression of genomes in the clade containing the two USA 2006 sequences (USA_2006; see Fig 1A) as well as their descendants. (D) Principal component analysis of genetic variants from the genomes in panel A. Each point is a genome colored by its geographic location. CDC, Centers for Disease Control and Prevention; MDPH, Massachusetts Department of Public Health. (TIF) [file pbio.3000611.s002.tif]

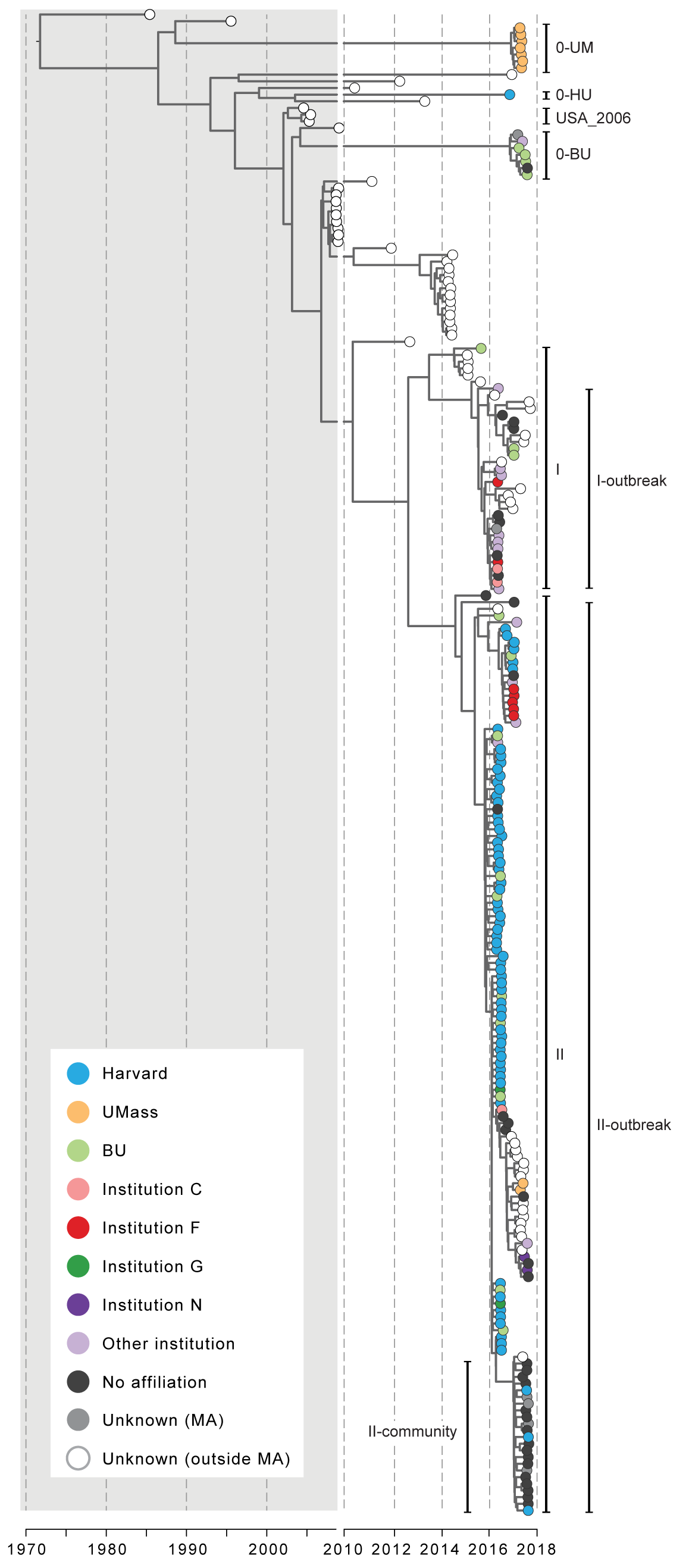

Supplement: S3 Fig — MCC tree of the 225 mumps virus genotype G genomes used in this study, colored by academic institution. Clades are labeled as in Fig 1A. MCC, maximum clade credibility. (TIF) [file pbio.3000611.s003.tif]

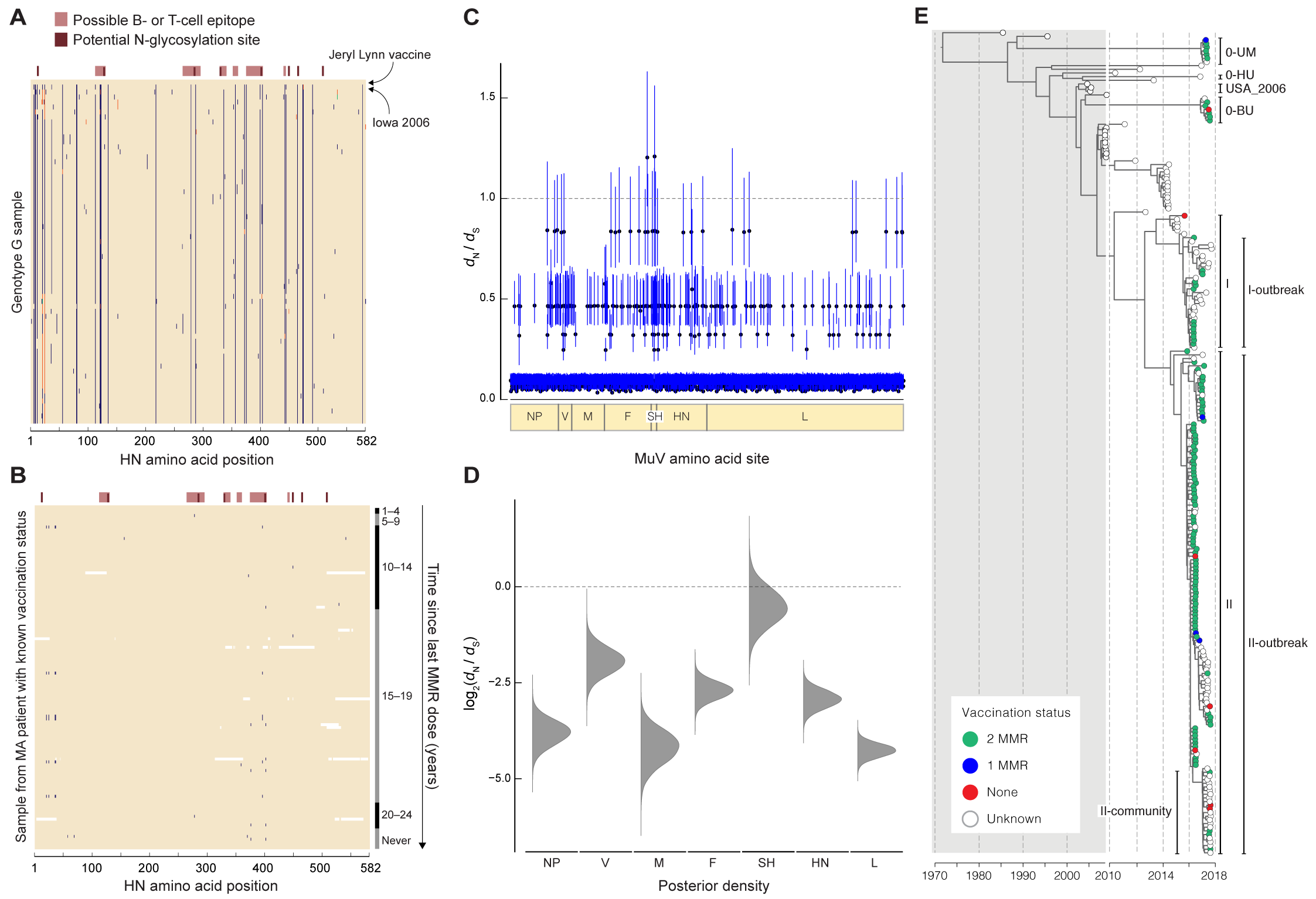

Supplement: S4 Fig — (A) Variation in genomes generated in this study. Each row represents one of the 119 mumps HN amino acid sequences from the individuals in our study who had known vaccine status. Samples are displayed in order of descending time since last MMR vaccine dose. Colored variants indicate variation from the consensus of all included sequences. (B) Variation in all published genotype G HN sequences. Each row represents one of the 456 publicly available mumps genotype G HN sequences (including from genomes generated in this study). Identical sequences are collapsed and then grouped by hierarchical clustering. In both panels, amino acid substitutions relative to the Jeryl Lynn vaccine strain are highlighted in blue, with orange indicating a second variant allele and green indicating a third. Light red bars indicate possible neutralizing antibody epitopes, and dark red bars indicate potential N-glycosylation sites. (C) Estimate of dN/dS at each amino acid site in MuV coding regions, calculated across all 225 genotype G genomes used in this study. At each site, the mean estimate and 95% credible interval (not corrected for multiple testing) is shown. (D) Posterior density of dN/dS in each gene, using the same data set. (E) MCC tree of the 225 mumps genotype G genomes used in this study, colored by vaccination status. Clades are labeled as in Fig 1A. HN, hemagglutinin gene; MCC, maximum clade credibility; MMR, Measles-Mumps-Rubella; MuV, mumps virus. (TIF) [file pbio.3000611.s004.tif]

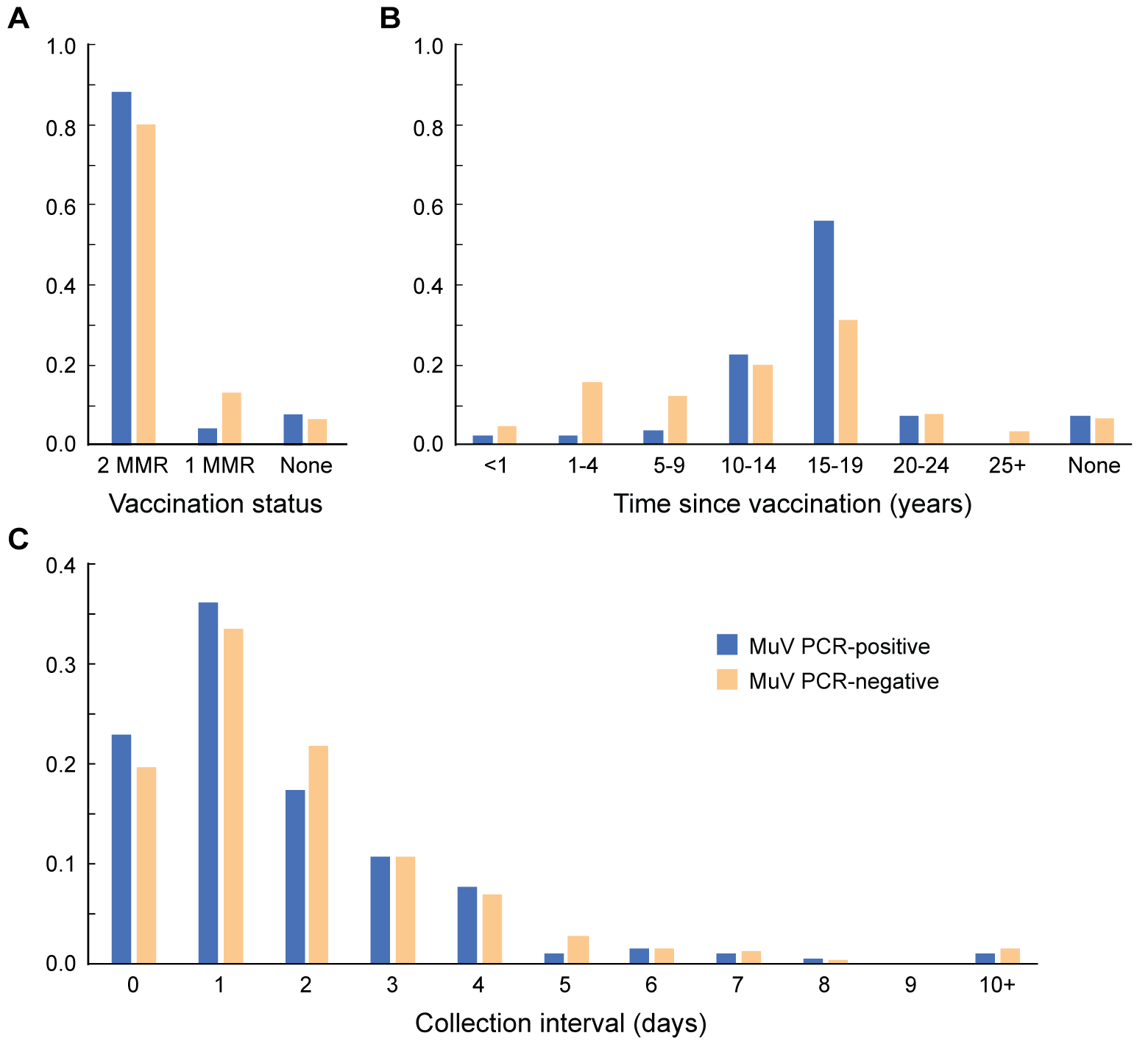

Supplement: S5 Fig — Comparison between 521 mumps PCR-negative samples tested in Massachusetts between 2016-01-01 and 2017-06-30, and 198 mumps samples from unique patients in Massachusetts in the same time period. In all panels, percentages have been recalculating after removing unknowns. (A) Vaccination status of positives (n = 198) and negatives (n = 309) with known vaccination status. A chi-square test suggests there is no relationship between PCR result and vaccination status (p = 0.012). (B) Years since vaccination for positives (n = 198) and negatives (n = 309) with known vaccination status. A chi-square tests suggests there is a relationship between PCR result and vaccination status (p = 1.19 × 10^−7^), and the plot shows that most recently vaccinated individuals were PCR-negative. (C) Collection interval for positives (n = 196) and negatives (n = 477) with known symptom onset and known collection date. A chi-square test suggests there is no relationship between PCR result and collection interval (p = 0.918). PCR, polymerase chain reaction. (TIF) [file pbio.3000611.s005.tif]

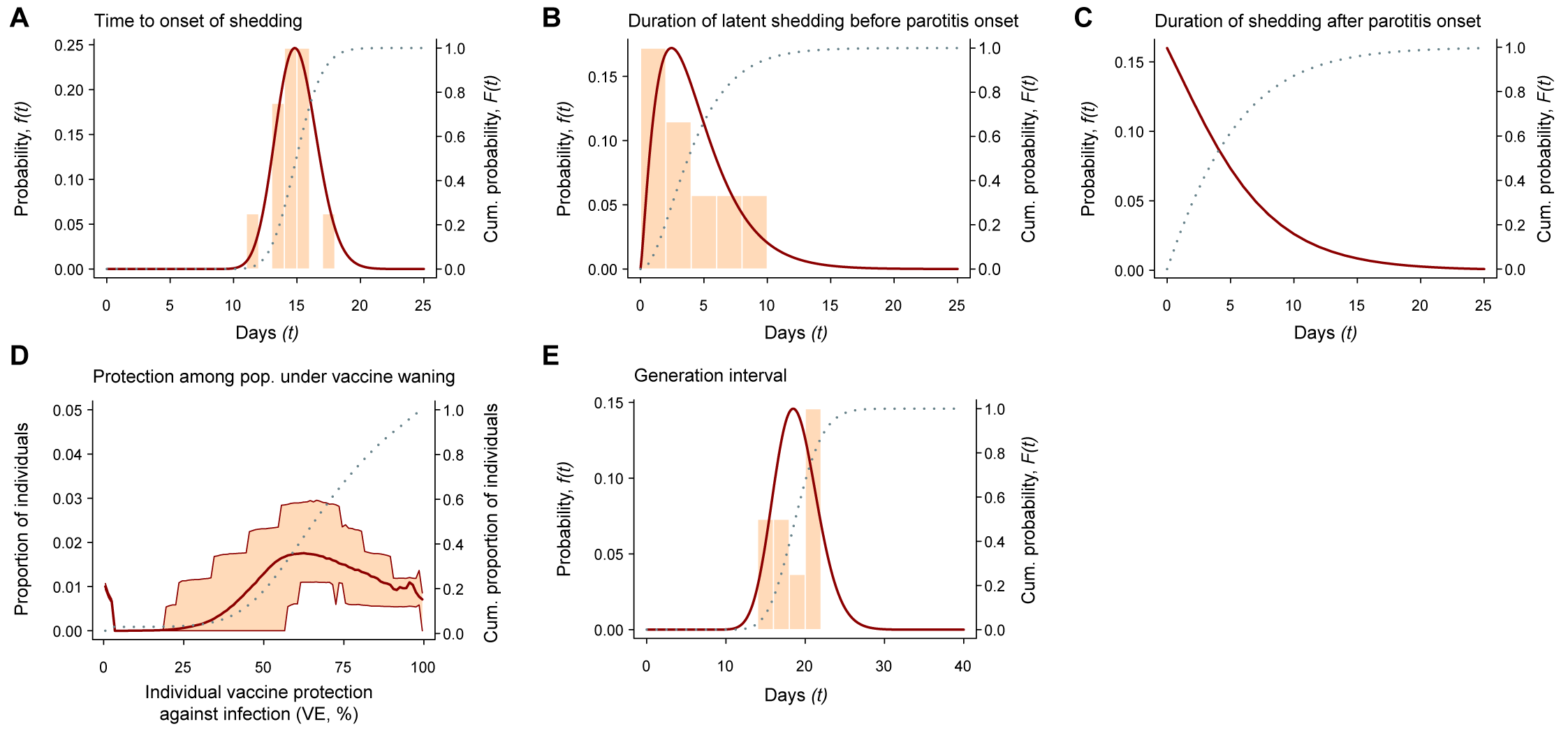

Supplement: S6 Fig — We illustrate fitted distributions of parameters of the modeled natural history of mumps infection. (A) We calibrate a gamma distribution to the duration of the incubation period—defined from the time of mumps virus exposure to the onset of shedding—using data from experimental human mumps virus infections with known exposure times [75]. (B) Onset of mumps shedding generally precedes onset of symptoms in the clinical course. We fit a gamma distribution describing the period of latent shedding to pooled data from 2 studies [75] and (C) apply previous estimates of the distribution of the duration of shedding after parotitis onset [77]. (D) We obtain estimates of the distribution of vaccine protection within a university protection by pairing previous estimates of the association between the strength of vaccine protection and time since receipt of the last dose [5] to data on vaccine coverage in a large university [80]. (E) We infer the distribution of the generation interval length in the Harvard using data from 10 cases with known exposure sources (“contact link”). A gamma distribution fitted by maximum likelihood recovers mean and dispersion estimates nearly identical to those reported in earlier mumps outbreaks [82]. (TIF) [file pbio.3000611.s006.tif]

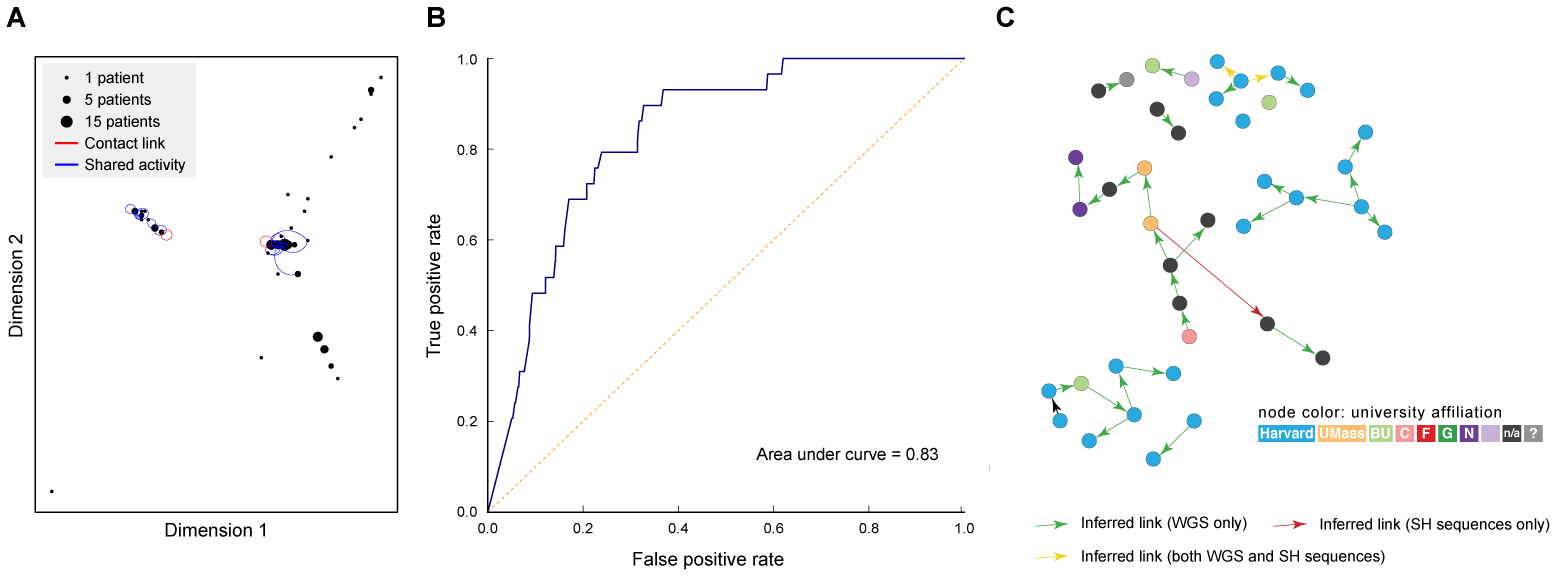

Supplement: S7 Fig — (A) Multidimensional scaling applied to samples in Clade II-outbreak (see Fig 1A). Each point is a mumps genome and pairwise dissimilarities are based on Hamming distance (see Materials and methods). Genomes with known epidemiological links are connected with a red line. (B) ROC curve for samples within Clade II-outbreak using pairwise genetic distance (calculated as in panel A) as a predictor of epidemiological linkage. (C) Transmission reconstruction using individuals within Clade II-outbreak, using the SH gene or whole genome sequences. Inferred links with probability ≥0.7 are shown; arrows are colored by data set. Nodes with one or more inferred links are shown and are colored by institution. See also Fig 2C, right. ROC, receiver operating characteristic; SH, small hydrophonic. (TIF) [file pbio.3000611.s007.tif]

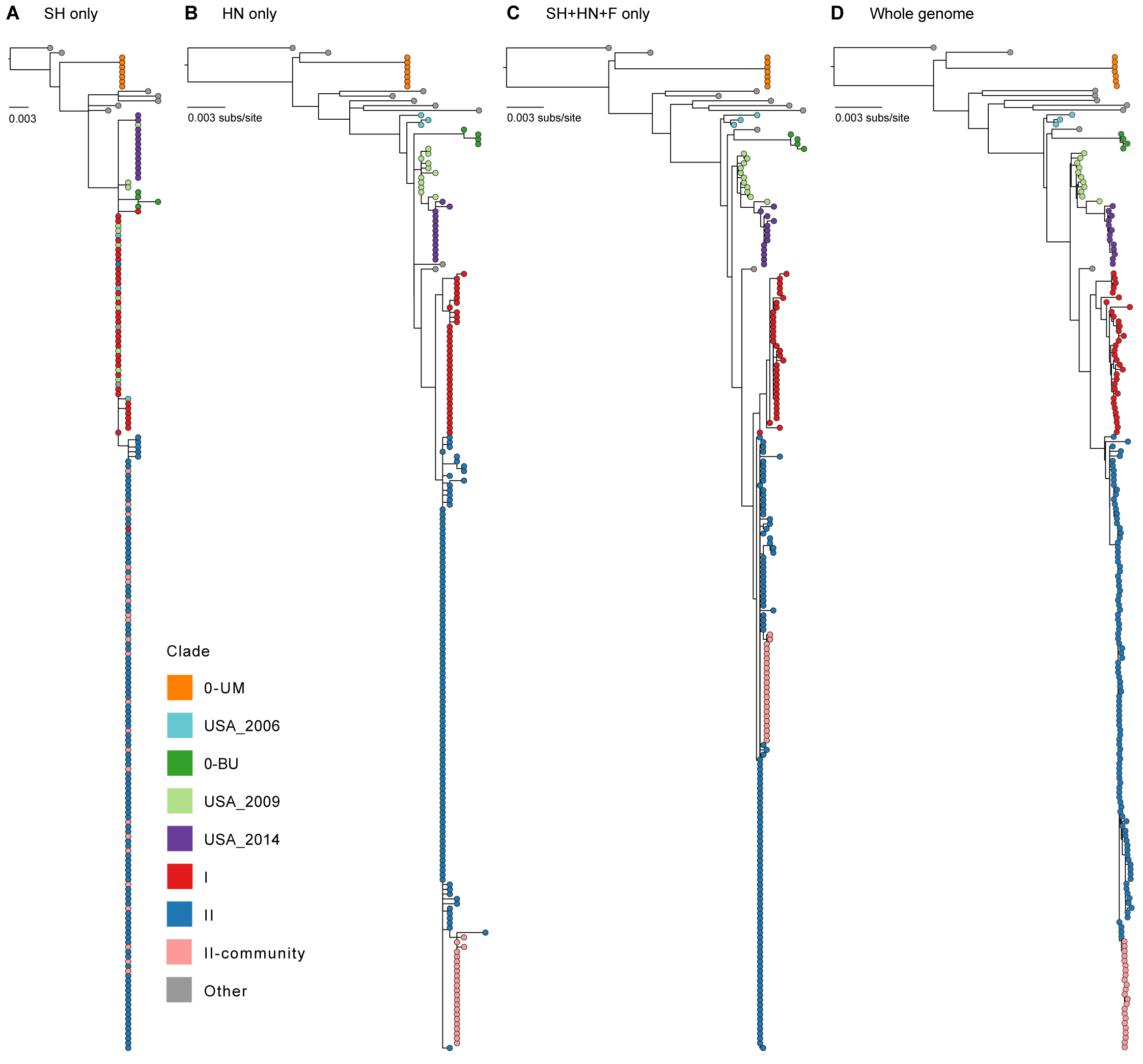

Supplement: S8 Fig — Maximum likelihood trees using (A) the SH gene only, (B) the HN protein only, (C) a concatenation of the HN protein, the F coding region, and the SH gene, and (D) the complete mumps genome. In all panels, tips are colored by clades as defined in Fig 1A and S2 Table. The HN protein sequence does a significantly better job at capturing the epidemiologically-relevant clades than the SH gene, and the tree created from SH+HN+F (nearly 25% of the genome) closely resembles the tree created from whole genome sequences. F, fusion protein; HN, hemagglutinin-neuraminidase; SH, small hydrophobic. (TIF) [file pbio.3000611.s008.tif]

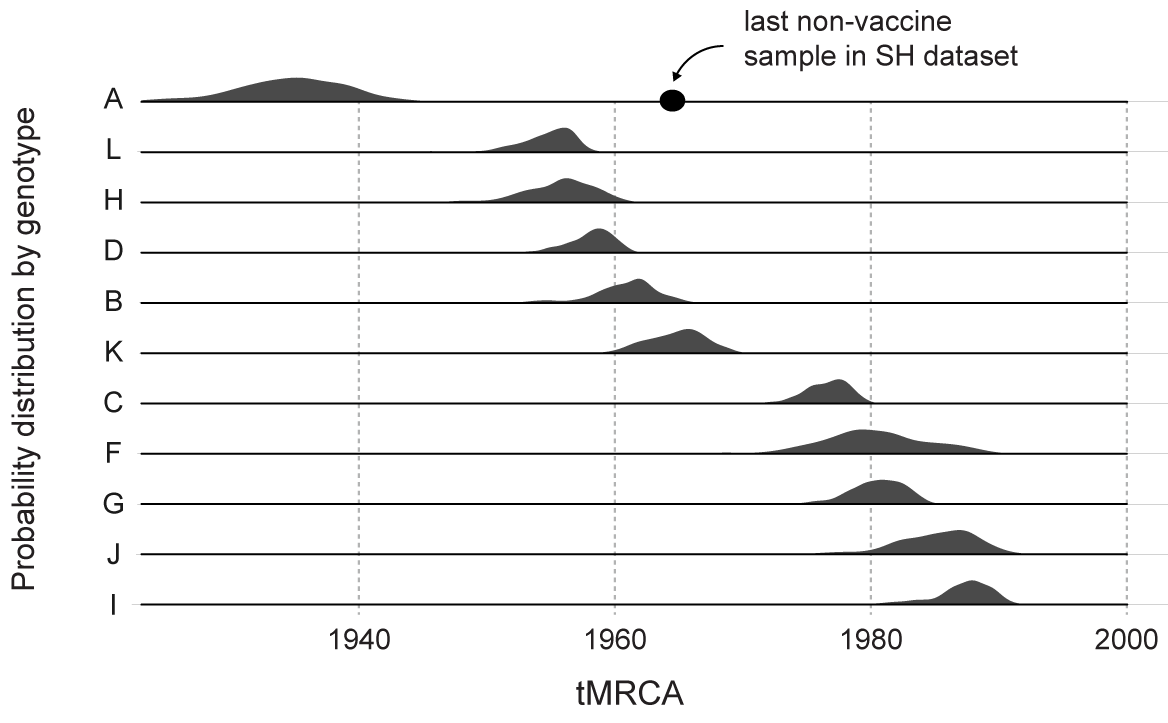

Supplement: S9 Fig — The date of the most recent genotype A clinical sample is indicated, excluding samples that closely resemble a mumps vaccine strain. SH, small hydrophobic; tMRCA, time to the most recent common ancestor. (TIF) [file pbio.3000611.s009.tif]

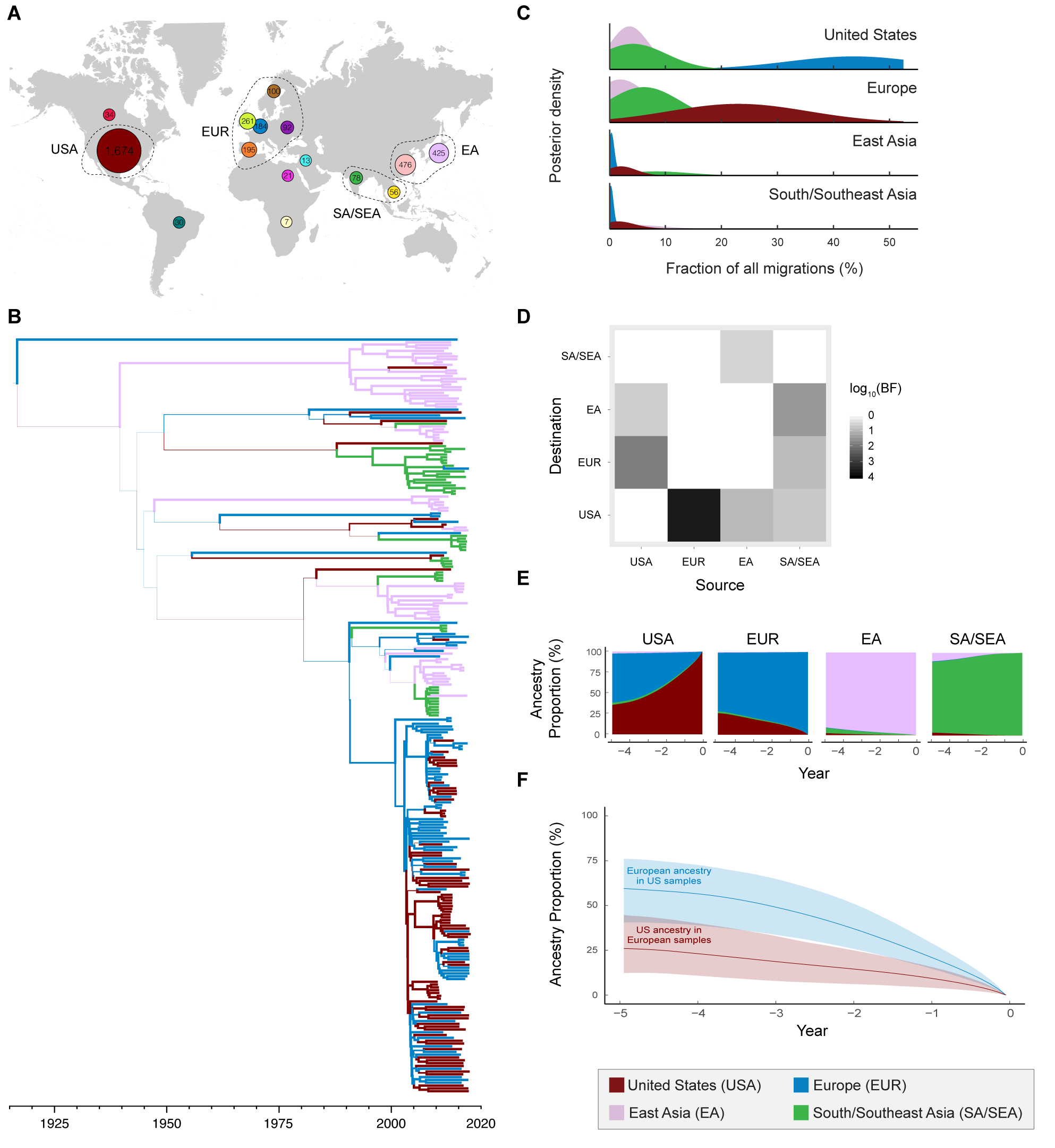

Supplement: S10 Fig — (A) World map indicating number of SH sequences in our data set from each of 15 regions; the 4 circled global regions represent the 4 regions from which we resampled input for migration analyses (see Materials and methods for details regarding geographic and temporal resampling of sequences). (B) Tree with the highest clade credibility across all trees generated on resampled input from 4 global regions. Branch line thickness corresponds to posterior support for ancestry (indicated by branch color). (C) Migration between the 4 global regions shown in panel A. Each plot shows a posterior probability density, taken across resampled input, of the fraction of all reconstructed migrations that occur to the destination (indicated in upper right) from each of the other 3 sources. (D) Migration between the 4 global regions shown in panel A. Shading of each migration route indicates its statistical support (quantified with BF) in explaining the diffusion of mumps virus. (E) Average proportion of geographic ancestry of samples in each of the 4 global regions (labeled) from each of the 4 regions (colored), going back 5 years from sample collection. (F) Average proportion of Europe in geographic ancestry of US samples, and vice-versa. Shaded regions are pointwise percentile bands (2.5% to 97.5%) across 100 resamplings of the input sequences. Colors for panels B,C,D,E,F are by global region, as shown in the bottom right. BF, Bayes factor; SH, small hydrophobic. (TIF) [file pbio.3000611.s010.tif]

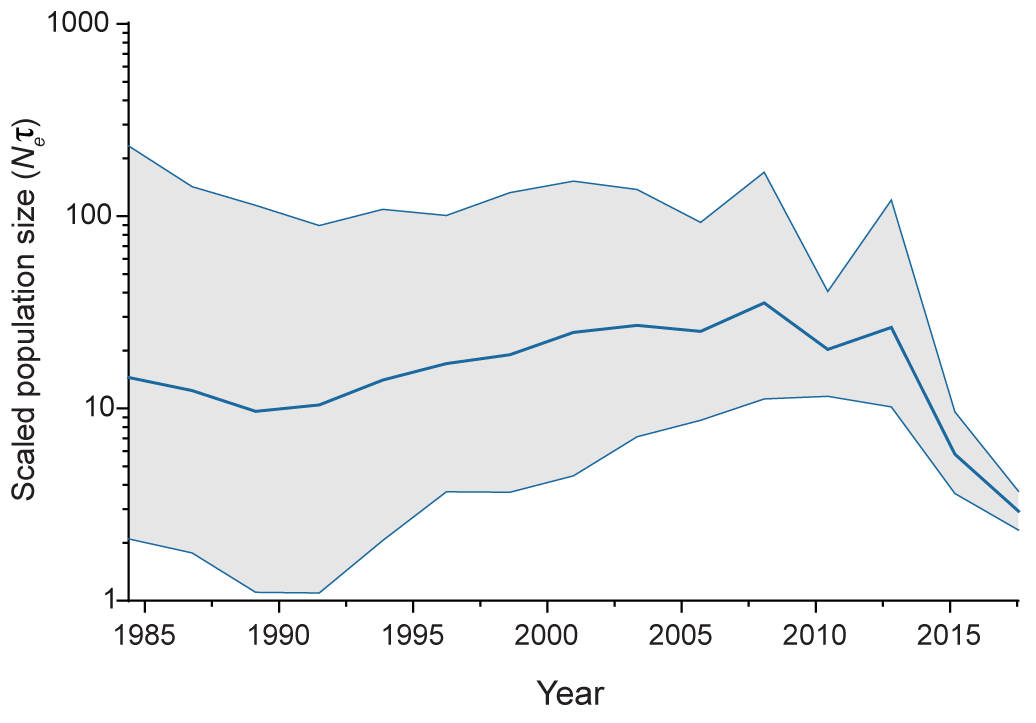

Supplement: S11 Fig — The scaled population size (Neτ) according to a Skygrid reconstruction. Dark line represents the median population size across samples from the posterior, and shaded area represents the 95% HPD interval. HPD, highest posterior density. (TIF) [file pbio.3000611.s011.tif]

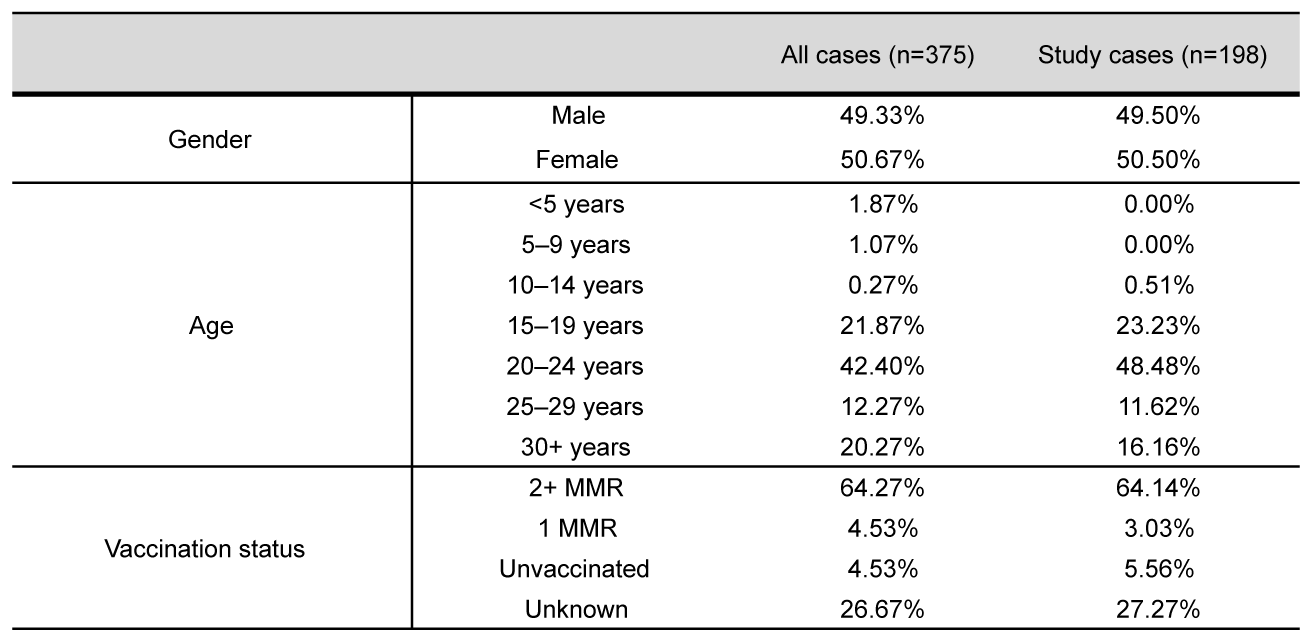

Supplement: S1 Table — Demographic information of all mumps cases in Massachusetts between 2016-01-01 and 2017-06-30, and the subset of these included in this study. (TIF) [file pbio.3000611.s012.tif]

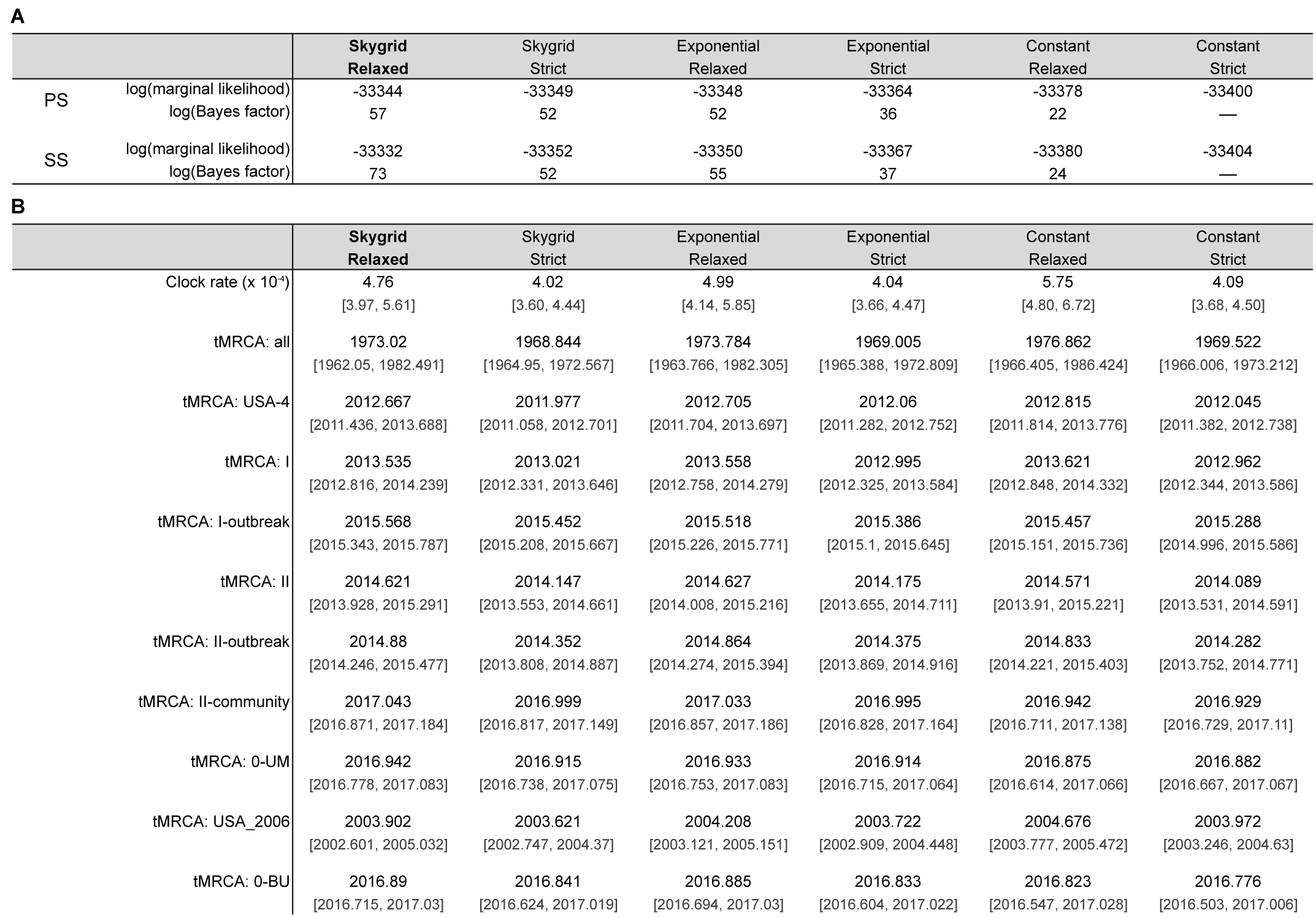

Supplement: S2 Table — (A) Marginal likelihoods estimated in 6 models: combinations of 3 coalescent tree priors (constant size population, exponential growth population, and Skygrid) and 2 clock models (strict clock and uncorrelated relaxed clock with log-normal distribution). Estimates are with PS and SS. The BF are calculated against the model with constant size population and a strict clock. (B) Mean estimates of clock rate, date of tree root, and tMRCAs of the clades shown in Fig 1A (excluding Clade 0-HU, which consists of one sample). USA-4 corresponds to “Clades I and II” in Fig 1A. Below each mean estimate is the 95% highest posterior density interval. tMRCAs of additional unlabeled nodes are available at http://doi.org/10.5281/zenodo.3338599. BF, Bayes factor; PS, path sampling; SS, stepping-stone sampling; tMRCA, time to the most recent common ancestor. (TIF) [file pbio.3000611.s013.tif]

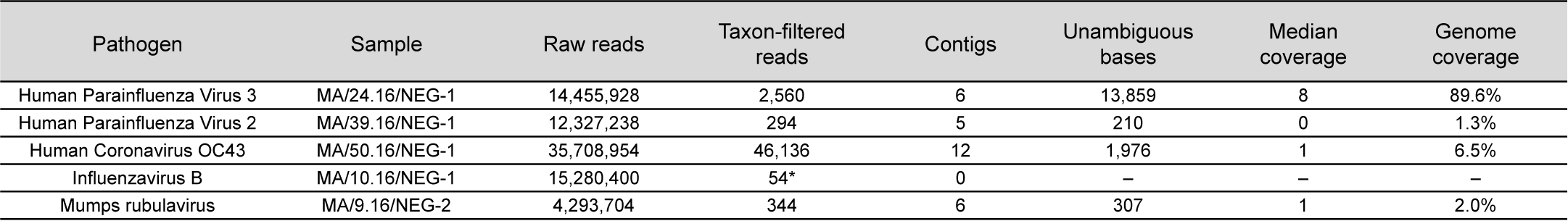

Supplement: S3 Table — Influenzavirus B is the only segmented virus listed, and we identify 51 reads mapping to 6 of the 8 segments: in order, 2, 6, 0, 0, 8, 10, 4, 21 reads to each segment. PCR, polymerase chain reaction. (TIF) [file pbio.3000611.s014.tif]
