# Supplementary material for: Combining genomics and epidemiology to track mumps virus transmission in the United States
Source: PLoS Biol. 2020 Feb 11;18(2):e3000611. doi: 10.1371/journal.pbio.3000611 (PMC7012397; doi:10.1371/journal.pbio.3000611)
Supplement: S1 Text — (DOCX) [file pbio.3000611.s019.docx]

**S1 Text**

Here we provide additional results and discussion about variants we identified in the mumps virus genome, and also describe our analysis of SH gene sequences from around the world.

***Variants potentially associated with vaccine escape***

There has been substantial interest in identifying factors that have contributed to the resurgence of mumps in the United States since 2006. There is increasing evidence for the role of waning immunity [1–3], which has recently led to the recommendation for a third vaccine dose in outbreak settings [4]. It remains unclear whether, or to what extent, there is also a contribution from virus evolution leading to escape from vaccine-induced immunity. This question is not straightforward to answer because there have been relatively few experimentally-defined mumps neutralizing antibody (NAb) epitopes [5–7], neutralization in vitro may not correlate well with clinical protection [8], and T cell immunity may play a role in protection from mumps virus [9,10]. Nevertheless, the extensive mumps genomic information generated in this study provided a unique opportunity to study possible mutations associated with vaccine escape.

Across the mumps genome, we did not observe any fixed nucleotide or amino acid substitutions among the individuals in this study who had no, incomplete, or distant MMR vaccination compared with those who had recent vaccination (**S4 Fig B** and **E**). We specifically examined amino acid substitutions in known immunogenic regions of the mumps genome. We used two reference sequences for variant calling: the Jeryl Lynn vaccine strain (accession: X93179) and a cell-passaged clinical strain isolated from Iowa in 2006 (accession: JX287385), near the beginning of mumps resurgence in the US. The Iowa 2006 strain was previously shown to be neutralized by sera from both vaccinated and naturally-infected individuals, albeit to a lower degree than neutralization of the Jeryl Lynn vaccine strain itself [3].

Within the 582-amino acid hemagglutinin protein (HN), which contains the major epitopes targeted by NAb [11], we observed 32 sites with fixed amino acid substitutions between our sequences and the Jeryl Lynn strain (**S4 Fig A**). Two of these amino acid substitutions occurred within experimentally-defined NAb epitopes (amino acid positions 336 and 356) [5–7]. Eight substitutions occurred in other regions potentially associated with neutralization escape (positions 113, 121, 122, 123, 375, 399, 403, and 442) [12], and three substitutions occurred in a putative NAb epitope identified in bioinformatic analysis (positions 464, 473, and 474) [13]. We also identified one loss (position 12) and one gain (position 464) of potential N-linked glycosylation sites, as well as two substitutions in bioinformatically-identified T cell epitopes (positions 279 and 287) [14].

All 32 of the sites with fixed amino acid substitutions in our sequences were also identified as variable positions in a recent analysis of HN sequences from the Netherlands [15]. In that study, the authors compared sequences from contemporary circulating genotype G viruses to historical sequences — primarily genotype A — to detect potential mutations associated with vaccine escape. The Jeryl Lynn vaccine strain belongs to genotype A, so the results of this investigation are relevant to our analyses described above. Gouma et al. identified four positions of interest that contained genotype-specific variants and occurred within potential NAb epitopes; three of these were also identified in our analysis (positions 113, 356, and 442), while the fourth (position 354) was conserved between our sequences and the Jeryl Lynn strain.

Notably, all of the 32 sites within HN with fixed amino acid substitutions were conserved between our sequences and the relatively neutralization-susceptible Iowa 2006 strain [3], except for positions 336 and 474: at position 336, the two previously-published Iowa sequences matched the Jeryl Lynn sequence, and at position 474, we observe two variants (one in Iowa sequences and one in Massachusetts sequences), neither of which match the Jeryl Lynn sequence. At these two positions, the variant observed in our sequences was also present in most other genotype G sequences published to date (**S4 Fig A**), including a recent study from the Netherlands [15]. This suggests that the Iowa 2006 sequence is not representative of most circulating genotype G viruses at these positions, perhaps due to cell passage, and further studies are warranted to test the neutralization susceptibility of strains containing these variants.

Separately, the nucleoprotein (NP) has also been demonstrated to contain NAb targets in its hypervariable hydrophilic C-terminus, specifically amino acids 412–475 and 475–549 [16]. In this region, we observed eight sites with fixed amino acid substitutions between our sequences and the Jeryl Lynn strain; there were no differences between our sequences and the Iowa 2006 sequence.

In total, we observed 40 fixed amino acid substitutions in immunogenic regions (32 in HN and eight in the C-terminus of NP) among our sequences compared to the Jeryl Lynn vaccine strain. Most of these were shared with the sequence of the relatively neutralization-susceptible Iowa 2006 strain, arguing against escape from vaccine-induced NAb during the 2016–2017 outbreak. However, further work is needed to test the neutralization susceptibility of strains containing L336S and V474A substitutions, which are represented in most circulating genotype G viruses but not the Jeryl Lynn vaccine strain or the neutralization-susceptible strain isolated from Iowa in 2006.

For completeness, we have also catalogued all variants in potentially immunogenic regions in HN and NP occurring in at least 3% of the 183 samples collected in the US during 2016–2017 (see **S2 Data**). These variants were not fixed in the population but may also be candidates for future functional studies.

***Recurring mutations***

Since mumps virus has only been exposed to selective pressure from the vaccine for a relatively short time, we considered the possibility that it is still evolving toward vaccine escape. In particular, we explored the possibility that the same advantageous mutations occurred multiple times in this outbreak. To this end, we searched for any sites that had evidence for recurring nonsynonymous mutations in our data and identified two. We observe three independent C>T (Ala>Val) mutations at nucleotide position 3,070 (based on a full 15,384-nucleotide mumps virus genome, e.g., accession JN012242.1; alternatively, amino acid 365 in the phosphoprotein, P). Nucleotide position 14,618 (AA 2061 in the polymerase, L) has an A (Thr) in most of the genotype G data, but a G (Ala) in the most basal branch (a sample from India, accession KF738113) and also in two subclades within the US. We present these mutations as possible candidates for future functional studies on mumps virus vaccine escape.

***Within-host variation and assessing the transmission bottleneck***

We attempted to use within-host variants (iSNVs) to better understand mumps transmission and evolution. We called iSNVs (see **Materials and Methods** for calling criteria) in 181 samples from our dataset (all 201 samples from unique patients, minus those sequenced in a batch with potential low-level contamination; see **Materials and Methods**). We identified at least one iSNV in 95 (52%) of these samples and hypothesize that low sequencing depth may contribute to the lack of iSNVs in some remaining samples. In total, across the 95 samples with at least one iSNV, we found 584 iSNVs that were present at >2% frequency.

To explore if iSNVs were transmitted between patients, we examined iSNVs in the five pairs of samples with known ‘contact links’ (see **Materials and Methods**) for which both patients in the pair have mumps genomes included in our final dataset (the consensus genome). In the 10 samples with known contacts for which we obtained a consensus genome, we identified at least one iSNV in only four samples, and did not identify any iSNVs shared between linked samples. More generally, we did not identify iSNVs above 2% frequency in any pair of samples, regardless of epidemiological linkage. Although we identified shared iSNVs at below 2% frequency in samples without known contacts, we did not find evidence that these shared iSNVs more often occurred in samples with more similar consensus mumps genomes, suggesting that these variants are not transmitted between individuals (or may be due to experimental and sequencing biases or errors).

The lack of shared iSNVs suggests that the mumps infection bottleneck may be too small to allow for transmission of low frequency variants. Alternatively, these data could also be explained by within-host dynamics that result in any transmitted iSNVs dropping below our limit of detection prior to sample collection. A better understanding of mumps within-host dynamics will be necessary to resolve these possibilities.

Within-host dynamics are often studied by comparing viral samples in a single patient at different stages of infection. Our dataset contains two individuals for which we have samples (and consensus mumps genomes) at two different time points, in both cases nine days apart. In both sets of samples, the later consensus genome differs from the earlier at one nucleotide position. In one patient, this variant is fixed in the later time point (i.e., it appears in all sequencing reads, at 73x read depth) and causes an amino acid change in the HN gene; in the other, there is an iSNV at the variant position, with 56% of reads matching the alternate allele (a synonymous variant in the L gene) and 42% matching the allele in the earlier sample (read depth = 55x) (2% have a third allele). There are several possible explanations for these observations — evolution and selective pressure within the host, sequestering of the virus within-host tissues or bodily compartments, sequential infection by two slightly different viral strains — but limited data (n=2) makes it difficult to do more than speculate. Future studies that reveal the nature of these within-host dynamics may prove useful in better understanding both mumps transmission and immune pressure within the host.

***SH genotype and phylogeographic analysis: findings and challenges in interpreting results***

We first calculated tMRCA for each of the 11 clinically relevant genotypes (**S9 Fig**) and found that genotype A, the strain used in most vaccines (including the Jeryl Lynn vaccine used in the United States), coalesces the earliest and appears to have stopped circulating (previously noted in ref. 17). The other genotypes, including genotype G, coalesce over a decade later.

We also performed a phylogeographic analysis on these SH sequences to understand movement of mumps between world regions (**S10 Fig A** and **B**). To reduce temporal and geographic sampling biases due in part to incomplete global mumps surveillance, we looked at samples collected since 2010 in four well-sampled regions (US, Europe, East Asia, and South/Southeast Asia). (For details regarding resampling, see **Materials and Methods**.) We find significant mumps migration between the US and Europe (**S10 Fig C** and **D**) and see that most introductions to the US are from Europe, although there is also support for mumps migrations from East Asia and South/Southeast Asia to the US. Likewise, most introductions to Europe appear to be from the US. We also find that recent sequences from the US have primarily European ancestry, and vice versa (**S10 Fig E** and **F**). Notably, recent sequences from East Asia and South/Southeast Asia have minimal external ancestry, suggesting relatively little spread to these regions (**S10 Fig E**).

*Demographic and clock models*: Whereas other demographic and clock models would have likely provided a better fit to the data than the one we used (see **Materials and Methods**), it appeared that using them (especially the use of a relaxed clock) would have made it impractical to achieve convergence and sufficient sampling of trees and all parameters on the full dataset.

*Phylogeographic models*: The full dataset has large temporal and spatial sampling biases that might affect phylogeographic results, such as estimates of migration; using a structured coalescent to model migration and coalescent processes could alleviate these biases, but might face practical limitations on this large dataset [18]. Instead, we used resampling on the input sequences (see **Materials and Methods** for details) to construct distributions of estimates.

*Limitations of resampling strategy*: Sampling biases affect this resampling strategy as well. Although the 4 global regions we used for resampling (US, Europe, East Asia, and South/Southeast Asia) encompass 97% of the SH gene sequences in our analysis, the resampling excludes large regions of the world from which sequences are unavailable or not well distributed temporally, and thus excludes migration to/from these regions. Relatedly, several years in the East Asia and the South/Southeast Asia regions have fewer than 10 sequences (sometimes, zero) available for resampling (**S4 Data**), which may lead to an underestimate on the relative rates of migration involving these regions. Moreover, the high sequence similarity between SH gene sequences from the United States and Europe (**Fig 3B**) may bias upward the distributions on the relative rates of migration between these regions and on the proportion of ancestry shared between them (**S10 Fig E**) (e.g., if there have been few true migrations between these regions, but the gene has not accumulated substitutions in the time between those migrations).

**References for S1 Text**

1. Cardemil CV, Dahl RM, James L, Wannemuehler K, Gary HE, Shah M, et al. Effectiveness of a Third Dose of MMR Vaccine for Mumps Outbreak Control. N Engl J Med. 2017;377: 947–956.

2. Lewnard JA, Grad YH. Vaccine waning and mumps re-emergence in the United States. Sci Transl Med. 2018;10: eaao5945.

3. Rubin SA, Qi L, Audet SA, Sullivan B, Carbone KM, Bellini WJ, et al. Antibody induced by immunization with the Jeryl Lynn mumps vaccine strain effectively neutralizes a heterologous wild-type mumps virus associated with a large outbreak. J Infect Dis. 2008;198: 508–515.

4. Marin M, Marlow M, Moore KL, Patel M. Recommendation of the Advisory Committee on Immunization Practices for Use of a Third Dose of Mumps Virus--Containing Vaccine in Persons at Increased Risk for Mumps During an Outbreak. MMWR Morb Mortal Wkly Rep. 2018;67: 33–38.

5. Kövamees J, Rydbeck R, Orvell C, Norrby E. Hemagglutinin-neuraminidase (HN) amino acid alterations in neutralization escape mutants of Kilham mumps virus. Virus Res. 1990;17: 119–129.

6. Orvell C, Alsheikhly AR, Kalantari M, Johansson B. Characterization of genotype-specific epitopes of the HN protein of mumps virus. J Gen Virol. 1997;78: 3187–3193.

7. Cusi MG, Fischer S, Sedlmeier R, Valassina M, Valensin PE, Donati M, et al. Localization of a new neutralizing epitope on the mumps virus hemagglutinin-neuraminidase protein. Virus Res. 2001;74: 133–137.

8. Peltola H, Kulkarni PS, Kapre SV, Paunio M, Jadhav SS, Dhere RM. Mumps outbreaks in Canada and the United States: time for new thinking on mumps vaccines. Clin Infect Dis. 2007;45: 459–466.

9. Gans H, Yasukawa L, Rinki M, DeHovitz R, Forghani B, Beeler J, et al. Immune responses to measles and mumps vaccination of infants at 6, 9, and 12 months. J Infect Dis. 2001;184: 817–826.

10. Jokinen S, Osterlund P, Julkunen I, Davidkin I. Cellular immunity to mumps virus in young adults 21 years after measles-mumps-rubella vaccination. J Infect Dis. 2007;196: 861–867.

11. Wolinsky JS, Waxham MN, Server AC. Protective effects of glycoprotein-specific monoclonal antibodies on the course of experimental mumps virus meningoencephalitis. J Virol. 1985;53: 727–734.

12. Šantak M, Lang-Balija M, Ivancic-Jelecki J, Košutić-Gulija T, Ljubin-Sternak S, Forcic D. Antigenic differences between vaccine and circulating wild-type mumps viruses decreases neutralization capacity of vaccine-induced antibodies. Epidemiol Infect. 2013;141: 1298–1309.

13. Kulkarni-Kale U, Ojha J, Manjari GS, Deobagkar DD, Mallya AD, Dhere RM, et al. Mapping antigenic diversity and strain specificity of mumps virus: A bioinformatics approach. Virology. 2007;359: 436–446.

14. Homan EJ, Bremel RD. Are cases of mumps in vaccinated patients attributable to mismatches in both vaccine T-cell and B-cell epitopes?: An immunoinformatic analysis. Hum Vaccin Immunother. 2014;10: 290–300.

15. Gouma S, Vermeire T, Van Gucht S, Martens L, Hutse V, Cremer J, et al. Differences in antigenic sites and other functional regions between genotype A and G mumps virus surface proteins. Sci Rep. 2018;8: 13337.

16. Tanabayashi K, Takeuchi K, Hishiyama M, Yamada A, Tsurudome M, Ito Y, et al. Nucleotide sequence of the leader and nucleocapsid protein gene of mumps virus and epitope mapping with the in vitro expressed nucleocapsid protein. Virology. 1990;177: 124–130.

17. Jin L, Örvell C, Myers R, Rota PA, Nakayama T, Forčić D, et al. Genomic diversity of mumps virus and global distribution of the 12 genotypes. Rev Med Virol. 2015;25: 85–101.

18. Müller NF, Rasmussen DA, Stadler T. The Structured Coalescent and Its Approximations. Mol Biol Evol. 2017;34: 2970–2981.
