# Supplementary material for: Combining genomics and epidemiology to track mumps virus transmission in the United States
Source: PLoS Biol. 2020 Feb 11;18(2):e3000611. doi: 10.1371/journal.pbio.3000611 (PMC7012397; doi:10.1371/journal.pbio.3000611)
Supplement: Attachment — Submitted filename: mumps-plosbio-response.docx [file pbio.3000611.s020.docx]

**Response to Referee #1**

*Wohl et al. provide an excellent investigation of the 2016-17 mumps virus outbreak in Massachusetts using genomic epidemiology. While I didn't have access to previous reviews and author responses, this version reads very well and tells a very interesting and complete story. The analyses all seem appropriate and the figures look nice and are mostly clear. I highly recommend this for publication and provide only a couple minor comments about the figures.*

We thank the reviewer for their thoughtful review and positive response to our manuscript.

*It wasn't until I read the main text did I really understand figure 2 (and I typically look at all of the figures before reading). There is a lot of important info buried in the legend, especially to explain B and C. Could the authors add a label to show that the left panels are estimates made from epi data and the right panels include genomic data? If this is clear from the start, the figure would be much clearer and more powerful.*

This is a good suggestion. We have updated Figure 2 to include labels indicating that the left panel data comes from epidemiological estimates only, while the right panel data adds genomic information.

*Given the space in the main text, I would recommend bringing some of the very nice and informative supplemental figures to the main text. The authors can choose, but I would recommend S1, S4 (but update the title to make it clear that this was used to investigate vaccine escape, then perhaps simplify it to showcase the main points), and S9.*

We are happy to hear this reviewer found the supplemental figures informative. We have moved the table in Figure S1 to the main text, since we felt that this summary of samples would be most useful to readers looking to understand our data. (It also addresses a comment from Reviewer #3 that it was hard to keep track of the number of genomes.) However, we have left the remaining panels in the supplement, since we wish to highlight the findings of our analysis rather than the sequencing process itself.

We opted to leave Figure S4 and S9 in supplementary material because there are limitations to these analyses, as detailed at length in supplementary text, and we believe keeping them alongside these details reduces a risk of over-interpretation.

**Response to Referee #2**

*Wohl et al. present a molecular epidemiological analysis of recent mumps outbreaks in Massachusetts during 2016-17. Using whole genome viral data, they are able to reconstruct the spread of mumps between neighboring communities including several universities in the region and connect these local outbreaks to larger-scale transmission patterns in the United States. They also explore whether mumps has undergone antigenic evolution to escape vaccine-based immunity, but do not find any genetic changes that can be correlated with vaccination patterns.*

*While this paper reports a perfectly valid molecular epidemiological analysis, the viral sequence dataset they analyze is limited to only ~200 samples collected mainly in MA from 2016-17.  Thus authors conclusions about mumps circulation in the larger U.S. are therefore somewhat suspect, and I believe the authors over interpret what such sparsely collected data can tell them (see main criticism below) about larger circulation patterns.*

*Results Lines 102-103: The authors find that a single mumps lineage has dominated in the US since 2006 and then argue that this “implies that continuous and extensive geographic movement of mumps virus, rather than isolated outbreaks, underlies mumps in the US” —>  I don’t actually see evidence for this claim in the phylogeny shown in Figure 1. If anything there is a fair amount of clustering in the phylogeny by region and lineages sampled in MA and NE one year are most closely related to lineages sampled in those regions the previous year. It’s therefore possible that the G genotype spread slowly throughout the US and the virus is now persisting locally in each region.*

We thank the reviewer for their feedback, and we would like to explain in more detail why we believe our data strongly supports extensive geographic movement of mumps in the United States. We would like to highlight that while a majority of these sequences are from Massachusetts, 43 genomes are from other states and span 15 other states that are geographically distributed around the country. These sequences comprise the largest and most geographically diverse whole genome mumps virus dataset from the US to date. Further, while we agree with the reviewer that denser sampling would clarify the picture of mumps transmission in the US, the 200 samples analyzed in this study represent ~50% of all mumps virus whole genome sequences available from anywhere in the world. We also note that recent studies with additional whole genome data (e.g., Alkam et al. 2019) support our findings.

Second, we realize that our presentation of the data and our oversampling of Massachusetts may have obscured the evidence for mumps virus mobility within the US. The key facts are these: We have numerous samples from two clades in Massachusetts. One of these, clade I, most likely has an MRCA in 2013 or later (see Figure 1C), and yet it contains samples from Massachusetts, from elsewhere in the Northeast, from the Midwest, and from the South. Clade II has an MRCA in 2014 or later, and yet it contains samples from all of those regions plus the West. Given our limited sampling of most of the country, finding such wide dispersal of closely related viruses is strong evidence of widespread geographic movement of mumps virus in the US on short timescales. To make this point more clear, we have extensively revised the text to more clearly lay out this argument and to highlight the MRCA estimates (Figure 1C and Table S2) that support it.

We would also like to point the reviewer to <https://nextstrain.org/mumps/na>, which contains published and unpublished mumps virus sequences from several studies, including ours. In this phylogeny, we see the same pattern as in our Figure 1 — clustering of samples from the same outbreak (which is inevitable, given that mumps virus can rapidly spread within a community), but the presence of recent samples from several regions of North America in every major clade of the tree.

*The possibility that mutations may have allowed for vaccine escape is of major interest, but no mutations were found to strongly correlate with vaccination status or show signs of positive selection (dN/dS > 1). But classic dN/dS ratios would be very underpowered to detect selection here (Kryazhimskiy et al, PLoS Genetics, 2008) since there has presumably not been enough time for the multiple mutations needed to produce a signal of positive selection under these tests to accumulate. In light of this, maybe it would be better to plot the frequency of each mutation among sampled viruses over time and look if any mutations in antigenic regions have dramatically increased in frequency?*

We agree with the reviewer’s comment that our dN/dS test is likely underpowered, despite our efforts to include the few available historical mumps samples (25 genomes from 1986–2016). We have rewritten the text in this section of the paper to caveat this analysis, and to explain why we attempted this analysis in light of limited historical sampling. We have elected not to plot the frequency of each mutation among sampled viruses over time, because the oversampling of recent samples from Massachusetts skews the frequency in a way that could be misleading. Instead, we have expanded on our analysis of mutations in antigenic regions that differ between the Jeryl Lynn vaccine strain and the sequences in this study (also shown in Figure S4).

*Line 544: ‘e-i(t)’ —> I’m guessing this should be e^{-lambda_i(t}?*

Yes, that is correct. Thank you for catching our mistake, we have fixed this in the text.

**Response to Referee #3**

*The authors generate approximately 200 new genomes of mumps virus circulating during an unusually large epidemic in and around Boston universities. They show that the virus consists of multiple widely circulating lineages. They highlight the increased resolution into transmission. Using the SH gene, they compare their sample to viruses circulating globally, and make the case for a persistent US-European lineage causing most cases. They demonstrate that the virus has an effective reproductive number RE well above one, and that control measures need to focus on blocking local transmission. A lot of work went into this impressively comprehensive analysis.*

*This study is well executed. The case that there is a lot of local transmission is convincing, and the finding of multiple closely related lineages circulating is surprising.*

*The authors also address the question of whether the virus has mutated to escape vaccine, and conclude that waning immunity is a more likely explanation for persistent transmission. I was less convinced by the robustness of this section of the paper. I was not convinced about the power of the dN dS analysis to detect escape from the vaccine selection pressure. At least, it was not clearly motivated what assumptions or hypotheses were being tested.*

We very much appreciate this reviewer’s constructive and detailed feedback. We have made substantial revisions to the vaccine analysis section of the paper, with special attention to more clearly laying out the hypotheses, rationales, and caveats of the tests we performed. The specific changes are detailed in the responses to the corresponding questions below.

*Perhaps more could be made of the data on time since vaccination, comparing negative and positive samples, and also formally testing whether subtitutions are associated with time since vaccination.*

See below for additional analysis comparing negative and positive samples. We also agree that more formal testing of the relationship between specific substitutions and time since vaccination (in addition to the analyses presented in S4 Fig B and E) would strengthen the arguments in this section. To do this, we focused on the mutations within our 2016–2017 samples (with known vaccination timing) in the HN gene. None had a frequency greater than 6% and they were largely present in the same samples. We therefore carried out a chi-square test on the one mutation in an immunologically-relevant region (see Figure S4) and also at an appreciable frequency (~5%), and found no association between presence/absence of that mutation and time since vaccination (p > 0.8).

*The data sharing statement should refer both to the sequences and to the associated metadata; currently the authors only propose to share sequences.*

Thank you for pointing this out. We have modified the data sharing statement to clarify that all of the associated metadata is available in Data S1 and in the BioSample submissions associated with the NCBI BioProject. We have also clarified that all of these metadata, as well as all genome sequences we generated, are already publicly available on NCBI.

Introductory paragraph, lines 46-56. How sure are the authors that temporally changing patterns of reporting might not affect these observations?

We have revised the text to acknowledge that changing clinical suspicion may have affected these observations, and that this background information reflects what was thought to be known about mumps transmission, rather than a true understanding of circulation patterns. That said, mumps has been a notifiable disease in the United States continuously since 1968 (see <https://wwwn.cdc.gov/nndss/conditions/mumps/>) — meaning that cases were required to be reported at either the state or national level — so we believe the general trends in incidence presented here to be accurate.

Line 60. ‘academic institutions and other close contact settings.’ Please use more specific language. What is a ‘close contact setting’? Does this include a household?

We have clarified what we mean by close contact setting by including examples with citations. Here we are referring specifically to populations where individuals have more day-to-day contact than average with people outside their household.

Line 60, and repeated several times later. Where possible, avoid open ended ranges such as ‘at least 18’, and instead use ranges and estimates.

We have clarified that mumps cases were reported to the Massachusetts Department of Public Health by exactly 18 colleges and universities, and have gone through the rest of the manuscript changing ‘at least’ to a specific range or estimate in eight different places. At least.

Line 65. I am not convinced that ‘phylogenomic analysis’ is a thing and think ‘phylogenetic analysis’ would be clearer.

Thank you for noting our error; we have changed the text to ‘phylogenetic analysis.’

Line 76. The number of genomes is variously 201 (here), 200 (line 96), and 203 (Line 220 and Figure S1). Please check to make this consistent, or specify if slightly different samples were used for different analyses.

We did indeed use slightly different samples for different analyses:

- We produced 203 mumps virus genomes, and thus report sequencing metrics on all 203 sequences.
- Two of these genomes were actually from a later time point sample from a patient already included in the study. Therefore, we have only 201 genomes from *unique* individuals, and refer to 201 genomes when presenting data associated with individuals, such as vaccination status.
- Of the 201 genomes from unique individuals, all but one belonged to genotype G (one was a genotype K genome), so our phylogenetic analysis includes only the 200 genotype G sequences.

We have revised the text in this section to present this information explicitly and in a more logical way, and have also added a new table to the main text (Table 1) that clearly shows these data.

Line 81. ‘Median sequencing depth’. Also specify 95% range.

We have added to the text the first and fourth quintiles of the sequencing depth, choosing these values to be consistent with Figure S1B. The 95% range is [8.1x to 8097.6x]. Readers can compute additional statistics from the line-level data in Data S1.

Line 81. ‘All genomes were at least 82% complete.’ This is not a fair description of the sequencing success rate, if I understand correctly the information provided in Fig S1. The authors generated 203 genomes from 259 samples. So 56 samples (22%) were excluded because of low viral loads associated with late collection of the samples. This is relevant information for readers interested in assessing the sequencing method used here. It is also possibly relevant as a sampling bias to consider in the discussion.

We have clarified this statement to refer only to the successful genomes: of the 203 genomes used in our analysis, each one had at least 12,614 unambiguous base calls, or 82% of the complete mumps virus genome. We also specified in the previous paragraph exactly how many samples were excluded (56, as the reviewer correctly points out), as we agree this gives readers a clearer understanding of the success of our sequencing methods. The new table added to the main text (Table 1), should also help highlight this information. We also now note in the text that the 56 excluded samples were well-distributed geographically and temporally, which should reduce concerns about the effect of sampling bias.

Lines 91-95. Whilst I am convinced that this conclusion is likely correct from the paper, I don’t see how you can conclude this from the analysis of the genomes (Figure 1). It rather seems to be a conclusion of the partial genes (Figure 3). To conclude this, you need to place the diversity in your sample in the context of the full global diversity, which you can only do with the SH gene. If you agree, then it seems you may need to present Figure 3 before Figure 1. I can see this is awkward since you want to showcase the genome trees & data, but you could make a case for the broad level information from a conventional SH analysis, and then zooming into the much higher resolution whole genome data.

We completely agree that the global diversity is necessary to make all the conclusions about mumps spread in the United States detailed in this paragraph. After much debate about the structure of the paper, we have opted to maintain the current narrative, since we feel that presenting the new data and findings first is most important. However, we have added a comment to the text explaining that single-gene data was used to support this finding, and mention that the SH analysis is discussed later in the paper. We hope this solution best addresses the reviewer’s legitimate concerns while not obfuscating the most interesting results from the study.

Lines 129-139. To me, this was the most opaque section of the paper. First on dN/dS: Why is a dN/dS analysis appropriate for addressing the question of vaccine escape? Why would vaccine selection produce higher dN than non-vaccine immunological selection?

As mentioned above, we have substantially revised this section of the paper to address the reviewers concerns, as well as those raised by Reviewer #2. In particular, we have highlighted that, although our genomic dataset was not particularly well suited to address questions of immune escape without experimental follow up, we explored many ways the variants in the data might be correlated with vaccination status. After explaining our analysis of variants in immunogenic regions, we then explain dN/dS as one test we used to identify sites under selective pressure.

We believe dN/dS analysis could be appropriate to address the question of vaccine escape because the virus has been circulating in the human population for a long time, while vaccination is relatively recent. This means that the virus could still be adapting to the vaccine, and that pressure from the vaccine is likely the largest selective force on the virus in the absence of widespread natural infection in the United States. However, our revised version is deliberate in stating that this test is likely underpowered, especially due to the small number (25) of historical samples collected prior to the 2016 outbreak.

Is the study powered for this comparison? It is not clear to me that a codon-by-codon analysis, as shown in Fig S4A or B is valid without correcting for multiple testing, and I don’t understand what conclusion, if any, the authors are drawing from this analysis.

We agree with the reviewer that this analysis is suboptimal. Our study is underpowered and does not explicitly correct for multiple testing, and we do not attempt to draw conclusions from this analysis. We included it because it is something that readers often want to see. In our revised manuscript we have pointed out the lack of correction.

In terms of the substitutions, the authors conclude that most mutations fixed here were already present in an ancestral sample (Iowa 2006), and thus that immune escape is unlikely (lines 991-998). The authors should make explicit the assumption of some kind of additive model here: why is not plausible that one of the two mutations identified, 336 and 474, could alone be responsible for vaccine escape? Without further wet-lab neutralisation assays, it must be hard to tell.

We agree with the reviewers that our text as written was not clear. To address these concerns, we have revised the text to be more explicit our assumptions and hypotheses regarding fixed mutations, moving some content from the supplementary discussion to the main text. To summarize: we assume that if vaccine escape was largely responsible for the outbreak, then the mutation must have happened early in the outbreak, and thus would be fixed in all the genomes we have generated. If vaccine escape happened between the 2006 and 2016 outbreaks, we would expect the responsible mutations to be fixed in the 2016 samples but not in the two from Iowa 2006. We agree with the reviewer that one of the two mutations that meet this criteria, at positions 336 and 474, could alone be responsible for vaccine escape.

We have also revised the text to highlight that neutralization assays have been done on the Iowa 2006 strain, and that this strain was only partially neutralization susceptible. In fact, a new paper (Rasheed et al 2019) was just published that suggested that the vaccine neutralizes the Jeryl Lynn strain 6-fold better than the circulating 2016 strain. We have updated our narrative to cite this new work, using this as evidence that one of the mutations we identified could be responsible for reduced efficacy of the vaccine in the currently circulating strain.

Overall, I can see why the authors did the analyses, the question is interesting. But the conclusions seem vague at this stage.

We agree that the conclusions from this analysis are rather vague, and thus have revised this section to keep it relatively short. We include the associated figures only in the supplementary material, where we can more clearly describe and caveat our results. We have also revised the text of the manuscript to more clearly spell out the caveats and limited conclusions that can be drawn, but believe that including even these speculative results could be useful for future experiments and analyses.

Lines 158-181. This is a convincing analysis, and really highlights the power of the whole genome data.

Thank you! We appreciate this positive feedback, and agree that this figure well conveys concrete epidemiological metrics that can be refined by adding information gleaned from whole genomes.

Methods. The authors use well-established in-house viral genomics methods and publicly available phylogenetic software. BEAST analysis: could you show the estimated skygrid plots for effective population size (Ne.tau)?

We agree this may be useful to many readers, and we have added a supplementary figure (S11 Fig) showing a Skygrid reconstruction of the scaled population size (*N_e_τ*). As a note to the reviewer, we caution against interpreting the recent decline, which could be an artifact of recent, dense sampling (PMC4989886).

PCA analysis (lines 478-481). Does this add anything to the paper?

We agree that the PCA does not add much to the paper not already shown by the phylogenetic tree. However, we believe some readers — in particular, those who come from outside this field — may appreciate this type of visualization, so we have elected to include it in the supplementary information. That said, we would be happy to remove the PCA plot if the editors feel it is unnecessary.

Stochastic model. The branching process approach looks sensible. The model estimates for lines 581-586 seem interesting, but I can’t see any estimates in the paper?

Thank you for pointing this out. We’ve clarified the protection values used from previous estimates. The estimate for necessary third-dose vaccine coverage, however, we have deliberately omitted at the request of the US Centers for Disease Control and Prevention, lest it be misconstrued as a recommendation.

Table S1. Please include the time since vaccination for both the positive and negative samples. Could more not be made of a comparison between the two?

We thank the reviewer for this suggestion. We have performed additional analyses, shown in a new supplementary figure (S5 Fig), which more clearly present the data and highlight the similarities and differences in the distributions of vaccine status, time since vaccination, and collection interval for negative and positive samples. As shown in the figures, there is very little difference in vaccine status or collection interval between the two groups. However, we found that there is in fact a relationship between mumps PCR result and time since vaccination (chi-square test p-value: 1.19e-7), with PCR-negative patients generally having more recent vaccination. These details are included in the legend of the new figure, and we have added this observation to the vaccine escape discussion in the main text. Thank you to the reviewer for encouraging us to do this analysis, which strengthens the argument that waning immunity may play a role in mumps infection.

Lines 1005-1010. This seems an orphan paragraph. Could you add a sentence to place these findings in context?

We have added text to the paragraph clarifying our motivation and explaining the possible significance of these findings.

Lines 1013-1045. These are interesting findings on lack of diversity in host, though paragraph 1035-1045 seems very speculative given the data.

We agree these findings are highly speculative, which is why we’ve chosen to include them only in the supplementary discussion. We were very intrigued by our finding that there was a change in the consensus genome between two time points from the same patient, but we did not have additional longitudinal data to extend these observations to a larger sample size. We elected to present the findings in case other readers find them interesting, but have updated the text to further emphasize how speculative the observation is. We hope future mumps studies have the necessary sampling to conduct a similar analysis with a larger sample size.
