# Supplementary material for: Combining genomics and epidemiology to track mumps virus transmission in the United States
Source: PLoS Biol. 2020 Feb 11;18(2):e3000611. doi: 10.1371/journal.pbio.3000611 (PMC7012397; doi:10.1371/journal.pbio.3000611)
Supplement: Attachment — Submitted filename: mumps-plosbio-response2.docx [file pbio.3000611.s021.docx]

**Response to Referee #2**

*The authors' revision has improved an already good paper. But I still disagree with their major conclusion that widespread geographic dispersal must be common.

Specifically, the authors say that: "Given the modest sampling in this dataset from outside the Northeast, finding such wide geographic dispersal suggests that long-distance migration of the virus is common in the US"

I hate to be difficult, but I really don't think this is the most parsimonious conclusion to be drawn from the phylogeny in Figure 1. Rather I would say there is strong evidence for regional circulation (e.g. in MA and the NE) with occasional long-distance dispersal to more distance regions of the U.S. After all, there is relatively strong clustering even from regions like the Midwest that were very under-sampled. Of course, more sampling from different regions may in fact reveal long-distance dispersal is common, I just don't feel that this should be assumed without evidence.*

We thank the reviewer for clarifying exactly which statements they take issue with, and for noting other possible conclusions. We agree that, while we have observed some evidence for long-distance dispersal, limited sampling of regions outside the Northeast means we cannot say definitively that this is a common event. We have updated this paragraph to reflect the speculative nature of our conclusion of long-distance migration, and to emphasize regional circulation with sporadic long-distance movement as an explanation. The paragraph in question now reads:

*The distribution of cases within the phylogenetic tree further suggests geographic movement of mumps virus on short time scales. For example, clade I (****Fig 1A****) contains virus genomes collected in 2015–2017 from Massachusetts, from elsewhere in the Northeast, from the South, and from the Midwest. The date of the most recent common ancestor of this clade was most likely in 2013 or later (****Fig 1C*** *and* ***S3 Table****), which implies that the virus spread to all of those regions in less than 5 years. Similarly, clade II contains genomes from all of those regions as well as one from the western US, all collected during the same time period. Clade II likely dates from no earlier than 2014, suggesting spread to these regions occurred in an even shorter amount of time. Together, these observations provide strong evidence for regional circulation in the Northeast, coupled with at least occasional movement to more distant geographic regions. Further sampling of these other regions may reveal additional long-distance dispersal events, which would indicate widespread geographic movement of mumps. This would not be surprising if transmission frequently occurs through college students, many of whom reside in close contact settings and also travel long distances to return home.*

Additionally, we have gone through the full text and toned down the language on “widespread” circulation, focusing on evidence for regional circulation and multiple introductions. We agree it is important to make sure our conclusions accurately reflect the data and thank the reviewer for pushing us to get this right.

**Response to Referee #3**

*The authors have done a great job responding the comments. They have strengthened their conclusions on waning immunity. They have clarified both the motivations for the different linked analyses, and highlighted limitations where appropriate. This is an impressively comprehensive study with several interesting and (to me, at least) unexpected conclusions.*

We thank you for your encouragement and for your very helpful and constructive reviews. They have certainly improved the paper and helped clarify both the methods of analysis and its limitations.
